# The False Recognition Test, a new tool for the assessment of false memories, with normative data from an Italian sample

**DOI:** 10.1007/s10072-024-07656-9

**Published:** 2024-06-17

**Authors:** Francesco Panico, Laura Catalano, Laura Sagliano, Luigi Trojano

**Affiliations:** University of Campania Luigi Vanvitelli, Viale Ellittico 31, 81100 Caserta, Italy

**Keywords:** False Memory, Aging, Dementia, Neuropsychology, Assessment

## Abstract

**Introduction:**

False memory can be defined as remembering something that did not happen. To a certain extent it is a normal phenomenon, but its occurrence seems to increase in healthy and pathological aging, possibly providing relevant clues on some clinical conditions in the spectrum of dementia. We adapted a well-established Deed-Roediger-McDermott paradigm, frequently used in experimental contexts, to devise a new neuropsychological assessment tool, the False Recognition Test (FRT), that can investigate classical facets of episodic memory performance (i.e. free recall and recognition), and assess proneness to produce semantically related and non-semantic false memories. Here we describe the FRT and provide normative data and correction grids to consider the possible effects of age, gender, and education on the FRT scores.

**Method:**

Two-hundred and thirty-two Italian healthy individuals (99 male) aged 18–91 years, with different educational levels (from primary to university) underwent the FRT, together with validated tests for cognitive screening and episodic memory assessment and one scale for depression.

**Results:**

Multiple linear regression analysis revealed that age and education significantly influenced performance on FRT. From the derived linear equations, we provide correction grids for the raw scores of the FRT, and equivalent scores estimated using a nonparametric method. Correlational analysis showed significant associations between FRT subscores and cognitive, executive and memory functions, and depression.

**Conclusion:**

The FRT may constitute a useful instrument for both clinical and research purposes.

**Supplementary Information:**

The online version contains supplementary material available at 10.1007/s10072-024-07656-9.

## Introduction

Since classical works, memory has been portrayed as a constructive rather than reproductive process, thus explaining possible pitfalls when recalling relevant information [1–3]. Among the possible errors related to episodic memory, production of false memories, i.e. the phenomenon of remembering information which was not given or an event that did not happen, has raised increasing interest. This has contributed in refining the theoretical models of memory [4, 5], and in bringing to light the possible implications of false memories in both clinical [6, 7] and forensic contexts [8, 9].

The Deed-Roediger-McDermott paradigm [DRM; 10] is a well-known procedure commonly used in experimental contexts to study false memory. This paradigm consists of learning lists of (often) verbal items, which are strongly semantically associated, and converge over a non-presented item known as the “lure”. For example, in the list “foot, heel, leather, walk”, the lure is “shoes”. When completing this task, individuals usually recall or recognise the lure, indeed not present in the list, and exhibit a high level of confidence of having been exposed to it during encoding. Some factors seem to modulate the occurrence of false memories as assed by the DRM paradigm, such as the associative strength between the words, and the emotional valence of the items [3].

In the clinical context, the systematic assessment of false memories has not received the same attention as the evaluation of forgetfulness. However, assessing false memories in neuropsychological patients could be as relevant as evaluating the decline of other memory processes, given the potential negative impact of erroneous memories on everyday life and independence. Indeed, although false memory formation and recall represent entirely normal phenomena, occurring at any age, the incidence of false memory seems to increase in both healthy aging [3] and pathological conditions such as Alzheimer’s Disease (AD) and amnestic Mild Cognitive Impairment (aMCI) [6, 7, 11–13]. Brueckner and Moritz [13] reported that both patients with aMCI and AD show higher false memory rates with respect to healthy people in an emotional version of the DRM paradigm. Chasles et al. [6] have recently investigated vulnerability to semantic and phonological interference in aMCI (as compared to healthy aging) by means of two word list learning tasks. The results showed an overall lower memory performance in aMCI, particularly susceptible to semantic interference (with a higher number of intrusion errors), than in healthy aging. Interestingly, other studies showed that false memory rate could differentiate between aMCI patients with and without β-amyloid plaques [14]. Thus, the study of false memory rates in neurological populations could be useful to identify new prognostic indices for predicting conversion from MCI to dementia. Moreover, the assessment of false memories could contribute to distinguish between different forms of dementia, such as dementia due to AD and the behavioural variant of Fronto-Temporal Dementia (bvFTD). Indeed, it has been proposed that a composite index and some qualitative cues based on the Rey-Auditory Verbal Learning Test (RAVLT) could differentiate AD and bvFTD [15, 16]. Relevantly, Ricci et al. [16] proposed that AD patients may be characterized by overall higher false memory rates, independent from the relation between encoded and recognition materials, whereas bvFTD patients may produce false memories limited to semantic content and less false memory rates when recognizing items not associated with the encoded targets.

The above studies raised interest on the relevance of assessing false memory in diagnosis and prognosis of MCI and dementia. However, to date no instrument is available specifically targeting false memories, i.e. no standardized, reliable, and valid psychometric neuropsychological tool allows to profile patients comparing recall, recognition, and false memory rates. To date, one tool that permits to assess semantic interference during memory recall is the Loewenstein-Acevedo Scale for Semantic Interference and Learning [LASSI-L; [17], no Italian version available]. The LASSI-L consists of presenting two lists of 15 words belonging to three semantic categories (fruits, clothes, and musical instruments); the participants are informed from the outset about the three categories which the words belong to, and are required to perform an immediate recall, a cued recall, and a delayed recall task. Another instrument is the Free and Cued Selective Reminding Test [FCSRT; [18, 19], which requires the encoding of items from different semantic categories, and evaluates immediate and late free and cued recall; this test provides a specific index assessing efficacy of semantic cues to facilitate retrieval from the stored information. However, both LASSI-L and the FCSRT have not been developed to specifically assess the production of false memories.

The aim of the present paper is to provide a new test for assessing false memories using the DRM paradigm. The False Recognition Test (FRT) involves encoding, immediate free recall, and recognition of lists of words controlled for associative semantic strength. Crucially, it allows to quantify semantic, non-semantic and total false memory rates. Here we also provide normative data from a large sample of healthy individuals accounting for the potential effects of gender, age, and education, and offering correction grids for evaluation of performance by newly tested individuals. The use of the FRT in neuropsychological practice may help diagnosis and clinical assessment of memory disorders in neurological populations.

## Methods

### Participants

Two-hundred and thirty-two healthy participants (99 male) aged 18–91 years (M = 44.53; SD = 21.45) with educational levels between 2 and 20 years (M = 12.97; SD = 4.17) were enrolled in the study. Participants were enrolled through acquaintances of the experimenters, workplaces, religious communities, senior centres, and advertisements on social media. All participants were community dwelling individuals living independently in several districts of Campania (South Italy) in urban and rural areas, and were either working or retired. We excluded individuals who reported current or history of alcohol or drug abuse, major psychiatric diseases, brain injury, stroke, dementia, or any other neurological illness. Moreover, all participants had to report good general health, although some frequent medical conditions associated with aging (hypertension or diabetes with a satisfactory drug treatment) were not considered exclusion criteria to avoid the selection of a “hyper-normal” group [20, 21]. All participants provided their written informed consent to participate in the study. Experimental procedures were approved by the Local Ethics Committee (approval number 20/2024). The distribution of the sample for age and educational level is described in Table 1.

**Table 1:** Demographics of the sample

	Age															
	18–30		30–39		40–49		50–59		60–69		70–79		80-		Tot	
Education	F	M	F	M	F	M	F	M	F	M	F	M	F	M		
1–5	-	-	-	-	1	-	-	-	2	-	8	1	8	3	23	
6–8	1	3	-	-	1	1	4	2	4	2	3	2	-	3	26	
9–13	17	22	1	5	8	1	12	4	3	8	6	3	2	-	92	
13-	31	23	7	3	1	4	10	6	3	2	1	-	-	-	91	
Tot	49	48	8	8	11	6	26	12	12	12	18	6	10	6	232	

### Procedure

All the participants completed a neuropsychological battery and filled in a questionnaire assessing their affective state. The tests are described below.

*Montreal Cognitive Assessment* (MoCA) [20] is a brief neuropsychological tool for screening global cognitive functioning. The test includes 8 sub-tests assessing: executive functions, visuo-spatial abilities, naming, short-term and long-term episodic memory, attention, lexical access, abstraction, spatial and temporal orientation. The test provides a maximum score of 30; the higher the score, the higher the level of global cognitive efficiency.

*Frontal Assessment Battery* (FAB) [21] is a screening neuropsychological battery formed by 6 sub-tests for evaluation of executive functioning: classification, mental flexibility, motor programming, sensibility to interference, inhibitory control and environmental autonomy. The total score is up to 18; the higher the score, the higher the level of executive functioning.

*Babcock Story Recall Test* (BSRT) [22] measures episodic verbal memory. It consists of an encoding phase in which the examiner verbally presents a short story, and an immediate and delayed recall phases where participants recall as many details as possible from the story. The maximum score is 16; the higher the score, the higher the memory performance.

*Beck Depression Inventory-II* [23, 24] is a self-report questionnaire with 21 items that measure depression levels. The scale is divided into two sub-scales, assessing somatic and cognitive depression, plus a total depression score. Higher scores at each scale indicate higher levels of depressive symptomatology.

*False Recognition Test* (FRT; see Supplementary File 1). The FRT is based on the DRM paradigm widely used to assess false memory in experimental contexts [3, 10]. The FRT is composed of three lists of semantically related words each specifically referring to a lure (victory, shoe, criminal) of different valence (one positive, one neutral, and one negative).

To develop the FRT, in a pilot study we selected fifteen critical target items from the Italian Affective Norms for English Words [25] intended to serve as possible lures. These target items were presented one-by-one to a wide sample of 117 healthy participants (36 males) not involved in the present study sample (age range 18–57, M = 29.5, SD = 7.4). The participants were asked to freely produce 5–7 words in relation to each target item. Then, we selected one critical lure for each valence (neutral, positive, and negative) among the target items eliciting most numerous and consistent responses; the 12 words most frequently reported by the participants in our sample were used to build the lists.

As a further step, we recruited an independent sample of 244 (79 males) healthy participants, not involved in the present normative study (age range 18–66, M = 26.7, SD = 11.3), who were required to evaluate the 3 lures and the words of the respective lists for valence, arousal, concreteness, and familiarity. Then, we chose three critical lures (one for each valence) with the highest association strength, and built an encoding and a recognition list related to each lure. In the FRT, each recognition list is formed by six words included in the encoding list and six words not presented during the encoding list (namely the critical lure, two strong semantically associated words and three not associated words, roughly balanced for familiarity and concreteness). Based on previous studies reporting a possible modulatory effect of some lexical variables on memory performance as assessed by the DRM and word recall and recognition paradigms [3, 26, 27], the encoding and recognition lists obtained using this procedure were controlled for associative strength, valence, arousal, concreteness, and familiarity (see Supplementary File 2). Although some differences in familiarity and concreteness were observed, the lists did not differ for association strength, while they differed for valence and arousal, consistent with our aims.

The procedure of FRT administration is as follows. During the encoding phase, the examiner reads each of the three lists one at a time (complete instructions are provided in Supplementary File 1). Thereafter, individuals are asked to freely recall as many words presented in the encoding phase. An interference period follows, in which individuals are asked to count from 1 to 10. In the subsequent recognition phase, a new list is presented, and the participants are asked to identify the words presented in the encoding phase in a list including the distractor words (i.e. words not present in the encoding list). The test provides six scores: Total Free Recall (score range: 0–36), Total Recognition (score range: 0–36), Total Failed Recognition (score range: 0–18), Total Semantic False Memory (score range: 0–9), Total Non-Semantic False Memory (score range: 0–9), Total False Memory (score range: 0–18). For free recall and recognition scores, the higher the score the higher the memory performance; for failed recognition, semantic false memory, non-semantic false memory and total false memory, higher scores mean lower memory performance.

### Data analysis

Descriptive statistics (mean, standard deviation, median and range) were obtained and correlation analyses between all the neuropsychological and affective measures were performed. Normative data, correction grids, and conversion in equivalent scores were performed following the procedure illustrated by Capitani [28] and Spinnler & Tognoni [22]. We first ran single regression analyses to assess the influence of gender (coded as male = 1; female = 0), age, and educational level (coded as years of schooling) on the participants performance on FRT subscales. Age and educational level (years of schooling) were entered in the regression models after applying logarithmic [log(100 – age)] and root square transformations respectively. Then multiple regression analyses were performed to evaluate any overlapping effect of age, gender, and educational level. The results of the multiple regression analyses were then entered into a regression equation to obtain a correction factor for each individual of the sample. Adjusted scores were obtained by adding or subtracting the contribution of concomitant variables from the original raw scores. Then the adjusted scores were ranked from the worst to the best using a non-parametric procedure, and a set of confidence at 95% was used to estimate limits of tolerance to identify “normal” or “abnormal” scores. Cut-off values were defined as the score at which or below which the probability that an individual belongs to the normal population is less than 0.05 [22, 28]. We then converted adjusted scores to a five-point interval scale (from 0 = score equal or lower than the 5% outer tolerance limit, to 4 = scores higher than the median value of the whole sample scores; 1 = score between the lower and inner tolerance limits; 2 and 3 obtained by dividing the area of distribution into two equal parts). Finally, a correction grid was built to allow adjustment of the raw scores of newly tested individuals for different possible combinations of age and education. All the statistical analyses were performed with SPSS v.25 statistic software.

## Results

Table 2 shows the descriptive statistics of demographics, MoCA, FAB, BRST, BDI-II and FRT scores. Table 3 shows the distribution of the scores at the FRT in the participant sample for each subtest.

**Table 2:** Descriptive statistics

	Mean	SD	Median	Range
Age	44.53	21.45	44	18–91
Education	12.97	4.17	13	2–20
MoCA adjusted score	23.07	3.32	23.46	16.15–30
FAB adjusted score	14.71	2.51	15.00	5.9–18
Episodic Memory	10.76	3.32	11.05	0–16.25
FRT				
*Free Recall*	17.49	4.88	18	0–29
*Recognition*	29.35	2.97	29	0–36
*Failed Recognition*	2.15	1.89	2	0–11
*Semantic False Memory*	4.24	2.02	4	0–9
*Non Semantic False Memory*	0.26	0.68	0	0–4
*Total False Memory*	4.50	2.35	4	0–11
BDI				
*Somatic*	9.40	5.28	9	0–25
*Cognitive*	4.68	4.41	3	0–23
*Total*	14.08	8.71	13	0–40

**Table 3:** FRT mean raw scores (± SD) by age and education

	Age							
Education	18–30	30–39	40–49	50–59	60–69	70–79	80-	Tot
*Free Recall*								
1–5	-	-	12.0	-	16.5 (2.1)	11.2 (4.3)	8.5 (2.0)	10.4 (3.8)
6–8	17.5 (1)	-	18.5 (4.9)	17.7 (3.0)	13.7 (5.0)	9.2 (4.6)	9.7 (2.9)	14.2 (5.1)
9–13	18.8 (3.5)	20.8 (4.7)	18.0 (4.6)	17.0 (2.9)	15.7 (4.2)	14.3 (3.6)	15.0 (8.5)	17.7 (4.1)
13-	20.5 (3.3)	22.1 (3.9)	19.4 (1.7)	18.6 (2.2)	16.4 (2.6)	15.0	-	20.0 (3.4)
Tot	19.7 (3.5)	21.6 (4.1)	18.1 (4.0)	17.8 (2.7)	15.4 (4.0)	12.1 (4.4)	9.5 (3.7)	17.5 (4.9)
*Recognition*								
1–5	-	-	28.0	-	29.5 (3.5)	25.9 (1.8)	24.8 (3.3)	25.8 (2.9)
6–8	29.5 (2.4)	-	28.5 (2.1)	28.7 (1.0)	27.7 (2.6)	26.4 (1.5)	25.7 (2.3)	27.8 (2.2)
9–13	30.5 (2.6)	30.5 (2.3)	29.3 (2.4)	29.6 (1.9)	27.9 (2.2)	27.3 (3.6)	28.0 (1.4)	29.6 (2.7)
13-	30.9 (2.1)	31.5 (2.5)	30.4 (1.5)	29.1 (2.9)	29.8 (3.3)	22.0	-	30.5 (2.6)
Tot	30.7 (2.3)	31.1 (2.4)	29.5 (2.1)	29.2 (2.2)	28.4 (2.6)	26.4 (2.7)	25.4 (3.0)	29.3 (3.0)
*Failed Recognition*								
1–5	-	-	1.0	-	1.5 (0.7)	3.3 (2.1)	2.9 (3.2)	2.9 (2.6)
6–8	2.0 (2.0)	-	4.0 (2.8)	2.3 (1.4)	2.3 (1.6)	4.4 (2.8)	5.0 (4.6)	3.1 (2.4)
9–13	1.9 (1.4)	1.3 (2.0)	2.2 (1.4)	1.6 (1.3)	3.2 (2.2)	2.8 (1.6)	3.0 (2.8)	2.1 (1.6)
13-	1.5 (1.4)	0.8 (0.8)	2.2 (1.3)	2.2 (2.2)	2.8 (2.2)	7.0	-	1.7 (1.7)
Tot	1.7 (1.4)	1.0 (1.3)	2.4 (1.5)	1.9 (1.7)	2.8 (2.0)	3.5 (2.1)	3.3 (3.3)	2.2 (1.9)
*Semantic False Memory*								
1–5	-	-	7.0	-	4.0 (2.8)	6.0 (1.7)	6.2 (1.8)	6.0 (1.8)
6–8	4.5 (2.1)	-	3.5 (0.7)	4.8 (1.2)	5.5 (1.9)	5.0 (1.6)	4.7 (2.5)	4.8 (1.6)
9–13	3.5 (2.0)	4.0 (2.3)	4.3 (2.1)	4.8 (2.1)	4.9 (2.1)	5.3 (2.2)	4.5 (2.1)	4.2 (2.1)
13-	3.5 (1.8)	3.8 (2.2)	3.4 (1.1)	4.6 (1.8)	3.2 (1.1)	4.0	-	3.7 (1.8)
Tot	3.5 (1.9)	3.9 (2.2)	4.1 (1.8)	4.7 (1.8)	4.6 (2.0)	5.5 (1.8)	5.7 (2.0)	4.2 (2.0)
*Non Semantic False Memory*								
1–5	-	-	0.0	-	1.0 (1.4)	0.8 (1.0)	2.0 (1.2)	1.3 (1.2)
6–8	0.3 (0.5)	-	0.0	0.2 (0.4)	0.5 (0.8)	0.8 (1.8)	0.7 (0.6)	0.4 (0.9)
9–13	0.1 (0.4)	0.0	0.2 (0.4)	0.1 (0.3)	0.0	0.4 (0.7)	0.5 (0.7)	0.1 (0.4)
13-	0.0	0.0	0.0	0.1 (0.3)	0.2 (0.4)	1.0	-	0.1 (0.2)
Tot	0.1 (0.3)	0.0	0.1 (0.3)	0.1 (0.3)	0.3 (0.6)	0.7 (1.0)	1.6 (1.2)	0.3 (0.7)
*Total False Memory*								
1–5	-	-	7.0	-	5.0 (4.2)	6.8 (2.5)	8.2 (2.7)	7.3 (2.7)
6–8	4.8 (1.7)	-	3.5 (0.7)	5.0 (1.4)	6.0 (2.6)	5.8 (2.8)	5.3 (2.5)	5.3 (2.1)
9–13	3.6 (2.1)	4.0 (2.3)	4.6 (2.2)	4.8 (2.2)	4.9 (2.1)	5.8 (2.5)	5.0 (1.4)	4.3 (2.2)
13-	3.5 (1.8)	3.8 (2.2)	3.4 (1.1)	4.7 (1.9)	3.4 (1.5)	5.0	-	3.8 (1.8)
Tot	3.6 (1.9)	3.9 (2.2)	4.2 (1.9)	4.8 (1.9)	4.9 (2.3)	6.1 (2.5)	7.3 (2.8)	4.5 (2.4)

Results from correlation analyses are reported in Table 4. Free Recall in the FRT was positively correlated with Recognition, educational level, MoCA, FAB, and BSRT and negatively correlated with Failed Recognition, Semantic False Memory, Non-Semantic False Memory, Total False Memory, age, and BDI scales. Recognition in the FRT was negatively correlated with Failed Recognition, Semantic and Non-Semantic False Memory, Total False Memory, age, and BDI scales, while it was positively correlated with educational level, MoCA, FAB, BSRT. Failed Recognition was positively correlated with Non-Semantic False Memory and age, and negatively correlated with educational level, MoCA, FAB and BSRT. Semantic False Memory was positively correlated with Non-Semantic False Memory, Total False Memory and age, and negatively correlated with educational level, MoCA and FAB. Non-Semantic False Memory was negatively correlated with gender, educational level, MoCA, FAB, BSRT and positively correlated with Total False Memory, age and BDI scales. Total False Memory was positively correlated with age and somatic and total BDI scales, while it was negatively correlated with education, MOCA, FAB and BSRT.

**Table 4:** Pearson correlations (r) and levels of significance for FRT scales, demographics, neuropsychological and affective measures

		1	2	3	4	5	6	Gender		Age	Education	MoCA	FAB	BSRT	BDI *Somatic*	BDI *Cognitive*	BDI *Total*
1. FRT *Free Recall*	r		0.544	-0.413	-0.290	-0.450	-0.378	0.020	-0.630		0.614	0.366	0.484	0.376	-0.250	-0.175	-0.240
	Sig		0.000	0.000	0.000	0.000	0.000	0.760	0.000		0.000	0.000	0.000	0.000	0.000	0.008	0.000
2. FRT *Recognition*	r			-0.591	-0.700	-0.572	-0.765	0.006	-0.567		0.479	0.272	0.317	0.308	-0.208	-0.137	-0.195
	Sig			0.000	0.000	0.000	0.000	0.922	0.000		0.000	0.000	0.000	0.000	0.001	0.038	0.003
3. FRT *Failed Recognition*	r				-0.103	0.134	-0.050	0.111	0.319		-0.239	-0.222	-0.183	-0.286	0.117	0.096	0.120
	Sig				0.117	0.041	0.450	0.090	0.000		0.000	0.001	0.005	0.000	0.074	0.146	0.069
4. FRT *Semantic False Memory*	r					0.368	0.963	-0.064	0.380		-0.329	-0.135	-0.204	-0.093	0.116	0.016	0.078
	Sig					0.000	0.000	0.335	0.000		0.000	0.040	0.002	0.159	0.079	0.803	0.234
5. FRT *Non Semantic False Memory*	r						0.604	-0.133	0.455		-0.483	-0.179	-0.321	-0.336	0.220	0.249	0.259
	Sig						0.000	0.043	0.000		0.000	0.006	0.000	0.000	0.001	0.000	0.000
6. FRT *Total False Memory*	r							-0.093	0.457		-0.421	-0.167	-0.267	-0.176	0.163	0.086	0.142
	Sig							0.159	0.000		0.000	0.011	0.000	0.007	0.013	0.192	0.031

In regression analyses, for Free Recall, Recognition, Semantic False Memory, Non Semantic False Memory, and Total False Memory the transformed value of age [log_(10)_(100-age)] and of education (square root of education years) were used. For Failed Recognition the transformed value of age [log_(10)_(100-age)] and the raw value of education were used.

The results from multiple regression analyses are shown in Table 5. For Free Recall, Recognition, Non-Semantic False Memory, and Total False Memory, the multiple regression analyses showed that the best models included age and education. The best model for Semantic False Memory included only age. The best model for Failed Recognition included age and gender.

**Table 5:** Multiple linear regression and beta unstandardised coefficients for the FRT scores

Measures	R	R^2^	F	p	B (unstandardised coefficients)		
					Age	Gender	Education
*FRT Free Recall*	0.705	0.497	113.113	< 0.001	10.23	ns	2.317
*FRT Recognition*	0.609	0.37	67.385	< 0.001	6.771	ns	0.693
*FRT Failed Recognition*	0.368	0.135	17.897	< 0.001	-3.099	0.601	ns
*FRT Semantic False Memory*	0.381	0.145	39.125	< 0.001	-3.553	ns	ns
*FRT Non Semantic False Memory*	0.583	0.339	58.805	< 0.001	-1.19	ns	-0.261
*FRT Total False Memory*	0.507	0.257	39.553	< 0.001	-3.837	ns	-0.698

ns = not significant

Formulas for exact direct calculation:

Adjusted Free Recall score = raw score – 10.23 x [log_(10)_(100—age) -1.69] -2.317 x [square root (year of education) -3.54]

Adjusted Recognition score = raw score – 6.771 x [log_(10)_(100—age) -1.69]—0.693 x [square root (year of education) – 3.54]

Adjusted Failed Recognition score = raw score + 3.099 x [log_(10)_(100—age) -1.69] – 0.601 x [if female = -0.42; if male = 0.58]

Adjusted Semantic False Memory score = raw score + 3.553 x [log_(10)_(100-age) – 1.69]

Adjusted Non Semantic False Memory score = raw score + 1.19 x [log_(10)_(100-age)—1.69] + 0.261 x [square root (year of education)—3.54]

Adjusted Total False Memory score = raw score + 3.837 x [log_(10)_(100-age) – 1.69] + 0.698 x [square root (year of education) – 3.54]

To allow adjustment of the raw scores of newly tested individuals according to demographic variables, a correction grid was built for the most frequent combinations of age (by 10-year steps) and educational level (according to the Italian schooling system). When the correction grid does not allow to adjust the raw score because individuals have demographic characteristics not included in the grid, we used extrapolation formula to estimate the adjusted scores. The correction factors for each subscale and conversion in equivalent scores are shown in Table 6.

**Table 6:** Correction grid for FRT scores according to demographic variables, conversion in equivalent scores, and cut off

Age								ES	Range
Education	18–30	30–39	40–49	50–59	60–69	70–79	80-		
*Free Recall*								0	< 11.36
1–5	0.88*	1.19*	2.67	3.65*	4.59	6.69	9.06	1	11.36–12.48
6–8	-0.29	-0.02*	1.30	2.05	3.13	4.58	6.27	2	12.49–15.22
9–13	-2.16	-1.62	-0.60	0.30	1.36	2.90	4.60	3	15.23–17.22
13-	-3.16	-2.93	-2.08	-0.94	-0.09	1.11	2.24*	4	> 17.22
*Recognition*								0	< 23.23
1–5	-0.51*	-0.31*	0.67	1.32*	1.94	3.12	4.69	1	23.23–26.25
6–8	-0.79	-0.61*	0.26	0.76	1.47	2.43	3.56	2	26.26–28.12
9–13	-1.31	-1.02	-0.34	0.21	0.87	1.87	3.04	3	28.13–29.44
13-	-1.65	-1.39	-0.78	-0.11	0.43	1.12	1.77*	4	> 29.44
*Failed Recognition*								0	> 6.22
F	0.61	0.24	0.35	0.13	-0.16	-0.55	-1.26	1	6.22–4.9
M	0.22	0.08	-0.22	-0.34	-0.73	-1.19	-1.47	2	4.8–2.87
								3	2.86–1.9
								4	< 1.9
*Total False Memory*								0	> 8.20
1–5	-0.11*	-0.22*	-0.78	-1.15*	-1.50	-2.24	-3.13	1	8.20–7.29
6–8	0.23	0.13*	-0.37	-0.65	-1.05	-1.60	-2.23	2	7.28–5.98
9–13	0.72	0.49	0.21	-0.11	-0.50	-1.08	-1.72	3	5.97–4.28
13-	1.07	0.89	0.66	0.25	-0.06	-0.50	-0.90*	4	< 4.28
*Semantic False Memory*						Any education		Cut off < 60 years	> 7
						Any education		Cut off ≥ 60 years	> 8
*Non Semantic False Memory*						Any education		Cut off < 60 years	> 1
						Education ≤ 5		Cut off 60–79 years	> 2
						Education > 5		Cut off 60–79 years	> 1
						Any education		Cut off ≥ 80 years	> 2

Note. * correction obtained by using extrapolation formula; for Failed Recognition no correction is needed for education, while adjustment is needed for sex (F = *female, M* = *male); Semantic and Non Semantic False Memory do not require correction thus only cut offs are provided*

For a sample of 232 subjects and using non-parametric procedure, outer and inner tolerance limits are defined by values corresponding to the 6th and 18th observations [28]. On this basis we estimated outer tolerance limits and Equivalent Scores FRT subtests (Table 6). When no correction factor was needed to adjust the raw score only cut-off scores are provided (Table 6).

## Discussion

The present work aimed at developing a new instrument, the FRT, for assessing false memories. The test adopted the procedure used in the experimental studies involving the DRM paradigm and controlled for associative semantic strength [10]. Moreover, as emotional valence has been suggested a possible modulatory factor when assessing false memory [3] the FRT included neutral, negative, and positive lists of words. The correlational analyses between the measures provided by the FRT and other neuropsychological and affective instruments offered relevant clues on the validity of the test. All the subscales of the FRT showed strong within-scales correlations, indicating internal consistency of the test. Older age was associated with significantly worse memory performance on all memory measures in line with previous evidence [3, 6, 29]. Similarly, education significantly affected performance on the FRT, with lower levels of education associated with worse memory performance, in line with previous studies on episodic memory [30–33]. Interestingly, it should be mentioned that education has been indexed within the multiply proxies of cognitive reserve, which seems to be associated with overall better cognitive and psychological functioning [33–35]. No relationship was observed between gender and most FRT subscales, but females produced a significantly higher number of non-semantic false positive answers. This seems to fit well with a recent study using the DRM paradigm showing that females had higher true and false recall rates than males [36].

Associations with cognitive tests (MOCA, FAB, and BSRT) showed that overall better performance on the FRT was correlated with higher cognitive functioning, better executive control, and better episodic memory performance. The positive correlation between our test and BSRT confirms the validity of our instrument in measuring memory performance and can be considered a proof of its convergent validity. The correlation between FRT subtests and FAB score might support a link between false memory and executive control. Indeed, these findings would fit one of the theories attempting to explain the nature of false memories as assessed by the DRM paradigm. According to the Activation Monitoring Theory [5], false memories could be related to the interaction between an implicit associative process, specific to the encoding phase, and a source monitoring process, activated during the recall phase. Within this theoretical framework, false recall can be described as a consequence of a failure in the source monitoring process leading individuals to mistake the internal associative activation of the critical encoded items for the external associative activation of new items presented during recognition phase [5]. We could speculate that the process of source monitoring as described in the Activation Monitoring Theory may rely on proper executive functioning, thus explaining the negative association between memory performance as assessed by the DRM paradigm and better executive control as measured by the FAB in our study. However, further studies are needed to clarify the relationships between executive functioning and false memory production. Finally, we found that higher levels of depressive symptoms were associated with worse performance at the FRT. This is in line with previous studies showing reduced cognitive functioning in individuals with depression [37, 38].

We also provided age-, education- and sex-stratified normative data for correction and interpretation of performance on the FRT, obtained from a sample of healthy individuals. To make the instrument suitable for use in clinical practice, we provided correction grids of raw scores for newly tested individuals. As the variance of the single subscale of the FRT was explained by different factors, we provided grids for each scale for raw score adjustment according to demographic factors.

We acknowledge two possible limitations in our normative and validation study which warrant attention by future work on this issue. First, we collected data from a large sample of individuals, with different sex, educational level and ages, from different districts, but all participants were recruited from the same region. Future studies could explore the need for region-specific norms as described for other neuropsychological tests [39]. A second possible limitation is related to the differences among the three lists in terms of familiarity and concreteness. We acknowledge that this difference might affect memory performance, but it should be noted that we adopted a data-driven approach selecting the words spontaneously produced by the experimental participants, thus not allowing a complete control on each lexical variable. Moreover, we believe that these differences are not particularly relevant for the present test, as it assesses memory performance considering the sum of scores from the three lists in a whole, thus reducing the weight of differences in familiarity among the lists in the subscores of the FRT.

The FRT could be used in neuropsychological assessment of patients with aMCI and dementia to obtain a more specific assessment of false memory impairment. Matias-Guiu et al. [40] compared the diagnostic accuracy of two other tests, the FCSRT and the LASSI-L, for the diagnosis of AD and aMCI using 18F-fluorodeoxyglucose positron emission tomography as a reference, showing that both instruments (with an advantage for the LASSI-L), are accurate in classifying patients with dementia. Thus, it would be relevant to validate the present new test in clinical populations. This could help identifying specific patterns of memory performance in clinical samples, thus contributing to an early diagnosis of cognitive decline as with other neuropsychological tools [40, 41]. Crucially the use of the present instrument could contribute to differential diagnosis among clinical pictures within the spectrum of dementia, such as dementia due to AD and fvFTD [15, 16], and to prediction of conversion from MCI to dementia [14]. Indeed as previously described by Ricci et al. [16] and recalled above, patients with AD may present overall higher false memory scores than patients with bvFTD, as the latter would produce false memory rates limited to semantic content of the material provided. Ricci et al. [16] also proposed a memory index, combining free recall, recognition, and false memory scores to differentiate AD and bvFTD. In a future validation study on AD and bvFTD patients, it would be interesting to adapt the index proposed by Ricci et al. [16] to the structure of FRT, to ascertain its specificity and sensitivity in diagnosing different forms of dementia. In line with these considerations, it would be worth investigating the neural correlates of false recognition as assessed by the FRT, also in ecological settings of evaluation [42], to comprehend functional and anatomical correlates associated with performance in the healthy and in the damaged brain [43]. Lastly, the FRT could help assessing susceptibility to false memory formation in the forensic context, as several studies have put on the spotlight the issue of false memories induction and of genuineness of relevant recalled episodes from witnesses and victims in forensic settings [8, 9]. Moreover, a specific variation of the FRT for forensic contexts could provide possible clues on malingering, by adopting some established approaches to detect malingering in forensic neuropsychological assessment (e.g. floor effect strategy and performance validity testing) [44].

## Supplementary Information

Below is the link to the electronic supplementary material.

Supplementary file1 (PDF 361 KB)

Supplementary file2 (DOCX 18 KB)

## Data Availability

Data will be provided upon request to the corresponding author.
