# Supplementary material for: The False Recognition Test, a new tool for the assessment of false memories, with normative data from an Italian sample
Source: Neurol Sci. 2024 Jun 17;45(11):5231–40. doi: 10.1007/s10072-024-07656-9 (PMC11470865; doi:10.1007/s10072-024-07656-9)
Supplement: Supplementary file 1 — Supplementary file1 (PDF 361 KB) [file 10072_2024_7656_MOESM1_ESM.pdf]

False Recognition Test (FRT)

Istruzioni. *"Le leggerò delle liste di parole che dovrà ascoltare con attenzione e poi ripetermi in qualsiasi ordine. A seguire le leggerò un'altra lista e per ogni parola dovrà indicarmi se era presente o meno nella lista subito precedente"*. [per ogni lista seguire lo schema Codifica – Rievocazione Libera – Conta 1-10 – Riconoscimento]

Conta 1-10

Lista 1 Codifica	Rievocazione Libera: Ordine	Intrusioni	Riconoscimento	Risp. (si/no)	Corretta (1/0)
Coppa			Podio (si)		
Premio			Trionfo (si)		
Squadra			**Vittoria** (no)		
Obiettivo			Traguardo (no)		
Podio			Primo (si)		
Successo			Squadra (si)		
Trionfo			Coppa (si)		
Trofeo			Gentilezza (no)		
Primo			Soddisfazione (no)		
Vincita			Sacrificio (si)		
Sacrificio			Occhi (no)		
Conquista			Vetro (no)		

Tot. __/12 Tot. __/12

Mancato Ric. __/6

Falso Ric. Semantico __/3

Falso Ric. Non Semantico __/3

Falso Ric. Tot. __/6

Conta 1-10

Lista 2 Codifica	Rievocazione Libera: Ordine	Intrusioni	Riconoscimento	Risp. (si/no)	Corretta (1/0)
Tacco			Posate (no)		
Suola			Volante (no)		
Calzini			Punta (si)		
Stivale			Lacci (no)		
Numero			Strada (si)		
Pelle			Piede (no)		
Correre			**Scarpa** (no)		
Stretta			Calzini (si)		
Punta			Numero (si)		
Alta			Fuoco (no)		
Strada			Correre (si)		
Bassa			Tacco (si)		

Tot. __/12 Tot. __/12

Mancato Ric. __/6

Falso Ric. Semantico __/3

Falso Ric. Non Semantico __/3

Falso Ric. Tot. __/6

Conta 1-10

Lista 3 Codifica	Rievocazione Libera: Ordine	Intrusioni	Riconoscimento	Risp. (si/no)	Corretta (1/0)
Omicidio			Manette (si)		
Reato			Carcere (no)		
Assassino			Omicidio (si)		
Pistola			Stanchezza (no)		
Pena			Ladro (no)		
Galera			Assassino (si)		
Furto			Pena (si)		
Rapina			Furto (si)		
Delinquente			Falso (no)		
Mafia			Fretta (no)		
Manette			**Criminale** (no)		
Passamontagna			Delinquente (si)		

Tot. __/12 Tot. __/12

Mancato Ric. __/6

Falso Ric. Semantico __/3

Falso Ric. Non Semantico __/3

Falso Ric. Tot. __/6

Mancato Ric. (1+2+3) __/18

Falso Ric. (1+2+3) Semantico __/9

Falso Ric. (1+2+3) Non Semantico __/9

Falso Ric. Tot __/18

Riev. Libera Tot. (1+2+3) __/36

Riconoscimento Tot. (1+2+3) __/36

Istruzioni False Recognition Test

Codifica. L'esaminatore legge la prima lista con una velocità di 1 parola al secondo.

Rievocazione Libera. L'esaminatore chiede al soggetto di ripetere liberamente le parole della lista appena presentata. Le parole possono essere ripetute dal soggetto in qualsiasi ordine. L'esaminatore prende nota dell'ordine con cui il soggetto ripete le parole e annota eventuali intrusioni negli spazi dedicati (ordine e intrusioni). Non è previsto alcun aiuto.

Conta da 1 a 10. Tra la rievocazione libera e il riconoscimento, l'esaminatore chiede al soggetto di contare dal numero 1 al numero 10.

Riconoscimento. L'esaminatore legge la lista di riconoscimento e per ciascuna parola chiede al soggetto se la parola presentata era presente o non era presente nella lista letta poco prima. L'esaminatore prende nota della risposta del soggetto e attribuisce il punteggio coerentemente.

Viene ripetuta la stessa procedura per la lista 2 e per la lista 3 (codifica, rievocazione libera e riconoscimento).

Attribuzione del punteggio

Per ogni lista viene calcolato:

- **Numero di risposte corrette alla rievocazione libera=** corrisponde al numero di parole correttamente rievocate dal soggetto contenute nella lista presentata subito prima.
- **Numero di risposte corrette al riconoscimento**= somma delle risposte in cui il soggetto ha risposto "si" quando la parola era effettivamente presente nella lista presentata prima e "no" quando invece la parola era assente.
- **Numero di mancati riconoscimenti**= somma delle risposte in cui il soggetto ha risposto "no" a una parola che era presente nella lista presentata (non ha riconosciuto una parola che c'era nella lista di codifica).
- **Numero di falsi ricordi semantici**= somma delle risposte in cui il soggetto ha risposto "si" ad una parola che non era presente nella lista di codifica per le parole semanticamente relate (per la lista 1: vittoria, traguardo, soddisfazione; per la lista 2: lacci, piede, scarpa; per la lista 3: carcere, ladro, criminale).
- **Numero di falsi ricordi non semantici=** somma delle risposte in cui il soggetto ha risposto "si" ad una parola che non era presente nella lista di codifica per le parole semanticamente non relate (per la lista 1: gentilezza, occhi, vetro; per la lista 2: posate, volante, fuoco; per la lista 3: stanchezza, falso, fretta).
- **Numero totale di falsi ricordi**= corrisponde alla somma dei falsi ricordi semantici e non semantici.

Viene poi calcolato il gran totale sommando i punteggi parziali alle tre liste (Lista 1+Lista2+Lista3).
