# Supplementary material for: The False Recognition Test, a new tool for the assessment of false memories, with normative data from an Italian sample
Source: Neurol Sci. 2024 Jun 17;45(11):5231–40. doi: 10.1007/s10072-024-07656-9 (PMC11470865; doi:10.1007/s10072-024-07656-9)
Supplement: Supplementary file 2 — Supplementary file2 (DOCX 18 KB) [file 10072_2024_7656_MOESM2_ESM.docx]

**Supplementary File 2**

*Control analyses on lexical variables for the lists of the False Recognition Test (FRT).*

The three lists of the FRT were compared for association strength, valence, arousal, concreteness, and familiarity using parametric repeated measures ANOVA or the non-parametric alternative (e.g. Friedman test), after checking for normality distribution of data by the Shapiro-Wilk test. Multiple comparisons were analysed using LSD post-hoc test or Wilcoxon test, where appropriate. All the statistical analyses were performed with SPSS v.20 software.

*Results for encoding lists.*

For association strength, Shapiro-Wilk test of normality revealed non-normal distribution for the neutral encoding list [W(12)=.831; p=.022], and normal distributions for positive [W(12)=.908; p=.203] and negative [W(12)=.901; p=.163] encoding lists, thus a non-parametric analysis was ran. Friedman test showed no difference between the encoding lists [χ^2^(2)=1.682; p=.431].

With respect to valence, data were normally distributed in the neutral [W(12)=.903, p=.175] and in positive [W(12)=.916; p=.258] encoding lists and non-normally distributed in the negative [W(12)=.808; p=.012] encoding list. Friedman test showed significant differences between the lists [χ^2^(2)=22.167; p<.001]. Wilcoxon multiple comparisons revealed that neural encoding list was lower in valence compared to positive encoding list [neutral median=5.65 SD=.69; positive median=7.53; SD=.69; Z=-2,981; p=.003] and higher in valence compared to negative encoding list [neutral median=5.65; SD=.69; negative median=1.70; SD=.65; Z=-3,059; p=.002], and positive encoding list was higher in valence compared to negative encoding list [positive median=7.53; SD=.69; negative median=1.70; SD=.65; Z=-3,061; p=.002].

For arousal, all the encoding lists showed normal distribution [neutral W(12)=.942; p=.529; positive W(12)=.951; p=.655 and negative W(12)=.890; p=.116]. Repeated measures ANOVA showed significant difference in arousal between the lists [F(2,22)=132,709; p<.001]. LSD post-hoc comparison showed significant difference between neutral [M=5.09; SD=.64] and negative [M=4.38; SD=.03] encoding lists [p=.013], neutral and positive [M=7.05; SD=.38] encoding lists [p<.001], negative and positive encoding lists [p=.013].

For concreteness, normal distribution in the neutral [W(12)=.888, p=.111] and in positive [W(12)=.880; p=.089] encoding list and a non-normal distribution in negative [W(12)=.786; p=.007] encoding lists were found. Friedman test showed no difference between the encoding lists [χ^2^(2)=2.000; p=.368].

For familiarity, all the encoding lists showed normal distribution [neutral W(12)=.961; p=.804; positive W(12)=.916; p=.253 and negative W(12)=.970; p=.913]. Repeated measures ANOVA showed significant difference between the encoding lists [F(2,22)=84.505; p<.001]. LSD post-hoc comparison showed significant difference between neutral [M=6.85; SD=.47] and negative [M=4.44; SD=.51] encoding lists [p<.001], negative and positive [M=6.67; SD=.45] encoding lists [p<.001], while no difference was found between positive and neutral encoding lists [p=.725].

*Results for recognition lists.*

Shapiro-Wilk tests for normality revealed non-normal distribution for the neutral list [W(11)=.846; p=.037], and normal distributions for positive [W(11)=.889; p=.134] and negative [W(11)=.93; p=.413] lists considering association strength. Friedman test showed no difference between the recognition lists [χ^2^(2)=.897; p=.639].

For valence, normal distribution for the neutral [W(12)=.961, p=.80], positive [W(12)=.899; p=.152] and negative [W(12)=.893; p=.13] recognition lists was found. Repeated measures ANOVA showed significant differences between the lists [F(2,22)=121.983; p<.001]. LSD multiple comparisons revealed that neutral recognition list was lower in valence compared to positive [M=5.71 SD=.55; M=7.27; SD=1.01; p=.002], and higher in valence compared to negative list [M=2.15; SD=.53; p<.001] and positive list was higher in valence compared to negative [p<.001].

For arousal, neutral and negative recognition lists showed a normal distribution [neutral W(12)=.949; p=.622; negative W(12)=.947; p=.588] while positive recognition list showed a non-normal distribution [W(12)=.838; p=.026]. Friedman test showed significant difference in arousal between the lists [χ^2^(2)=17.167; p<.001]. Wilcoxon post-hoc comparison showed significant difference between neutral [median=5.21; SD=.77] and negative [median=4.47; SD=.54] recognition lists [Z =-2.275; p=.023], neutral and positive [median=6.95; SD=.84] recognition lists [Z=-2.981; p=.003], negative and positive recognition lists [Z=-3.059; p=.002].

For the concreteness, a normal distribution for positive [W(12)=.90; p=.157] recognition list and a non-normal distribution for neutral [W(12)=.841; p=.028] and negative [W(12)=.777; p=.005] recognition lists was found. Friedman test showed significant difference between the recognition lists [χ^2^(2)=6.167; p=.046]. Wilcoxon post-hoc comparison showed significant difference between neutral [median=7.13; SD=.92] and positive [median=5.44; SD=1.31] recognition lists [Z =-2.118; p=.034], while no differences were found between neutral and negative recognition lists [Z=-1.883; p=.06], and negative and positive recognition lists [Z=-.392; p=.69].

For familiarity, all the recognition lists showed normal distributions [neutral W(12)=.950; p=.64; positive W(12)=.963; p=.82 and negative W(12)=.906; p=.18]. Repeated measures ANOVA showed significant difference between the recognition lists [F(2,22)=24.949; p<.001]. LSD post-hoc comparison showed significant difference between neutral [M=7.05; SD=.52] and negative [M=5.17; SD=1.22; p=.001] recognition lists, negative and positive [M=7.00; SD=.56; p<.001] recognition lists, while no difference was found between positive and neutral recognition lists [p=.768].
